# Spatial and temporal immune landscapes of non-necrotic granulomas in two murine tuberculosis models with divergent disease outcomes

**DOI:** 10.3389/fimmu.2026.1821629

**Published:** 2026-08-13

**Authors:** Malik Zohaib Ali, Taru S. Dutt, Johnathan Patterson, Angela Minic, Kimberly R. Jordan, Sara E. Maloney Norcross, Bernd Meibohm, Mercedes Gonzalez-Juarrero

**Affiliations:** 1 Mycobacteria Research Laboratories, Colorado State University, Fort Collins, CO, United States; 2 Microbiology, Colorado State University, Immunology and Pathology, Fort Collins, CO, United States; 3 Program in Cell & Molecular Biology, Colorado State University, Fort Collins, CO, United States; 4 Department of Pathology, Heersink School of Medicine, University of Alabama at Birmingham, Birmingham, CO, United States; 5 Department of Immunology and Microbiology , University of Colorado Anschutz Medical Campus, Denver, CO, United States; 6 Engineering and Advanced Technology Department, RTI International, Durham, NC, United States; 7 Department of Pharmaceutical Sciences, University of Tennessee Health Science Center, Memphis, TN, United States

**Keywords:** BALB/c mouse model, C3HeB/FeJ mouse model, immune cells, inForm Tissue Finder, multiplex fluorescence immunohistochemistry, *Mycobacterium tuberculosis*, non-necrotic granuloma, spatial analysis

## Abstract

Tuberculosis (TB), caused by *Mycobacterium tuberculosis*, continues to be a major health issue worldwide. Granulomas are characteristic lesions of TB, functioning both as host defense structures and as sites where bacteria persist. To explore immune features associated with bacterial control versus disease progression, we examined the cellular composition and spatial arrangement of non-necrotic granulomas in two mouse models with distinct immune responses and outcomes: Balb/c mice, which partially control infection, and C3HeB/FeJ mice, which develop high bacterial loads and extensive pathology. Using multiplex immunohistochemistry, digital pathology, and spatial analysis, we measured immune cell densities, phenotypic diversity, and cell-to-cell spatial relationships, including median nearest-neighbor distances within granulomas over time. Balb/c granulomas showed higher cell density, greater immune diversity, and increased interactions between CD4+ T cells and macrophages, all of which are markers of effective IFNγ-dependent macrophage activation. These mice also exhibited early accumulation of Foxp3+ regulatory T cells and IL-10+ cells, followed by controlled infiltration of CD8+ T cells and Ly6G+ neutrophils, correlating with a reduction in lung bacterial burden. Conversely, C3HeB/FeJ granulomas were larger but less dense, dominated early on by Ly6G+ neutrophils, and revealed increased median nearest-neighbor distances between CD4+ T cells and macrophages, despite ongoing immune cell presence and bacterial loads. Our comparison of non-necrotic granulomas across both models indicates that granuloma size alone does not determine bacterial control. Instead, differences in immune cell composition, spatial arrangement, and specific cell interactions distinguish granulomas in mice without evident disease progression from those showing disease progression. These results suggest that spatial immune architecture is a crucial factor influencing the outcome of TB disease.

## Introduction

Tuberculosis (TB) remains the leading cause of death globally from a single infectious agent (1–4). It results from infection with *Mycobacterium tuberculosis* (*Mtb*), mainly transmitted through aerosolized droplets expelled during breathing and coughing. Clinical presentations range from asymptomatic latent infection—where about 90% of individuals harbor bacteria within granulomas—to active TB, which involves progressive lung damage, necrosis, and potential transmission. Host factors such as immune response, genetics, health, and environment influence disease susceptibility and progression (5–8).

A key pathological feature of TB is granuloma formation—organized clusters of multiple immune cells that develop when macrophages cannot eliminate *Mtb* (9–11). In the lungs, these granulomas are highly diverse. Studies of human lung tissue and animal models show significant variability in structure, cellular composition, and development, indicating that each granuloma forms independently and is influenced by local immune activity, bacterial load, and host physiology (12, 13). Mature granulomas contain various macrophage-derived cells, including epithelioid macrophages, foamy macrophages, and multinucleated giant cells, as well as T cells, B cells, neutrophils, and other leukocytes (14, 15). Cytokines such as interferon-gamma (IFNγ) are vital for activating macrophages’ antimicrobial functions and controlling bacterial growth (10, 16).

Although granulomas are meant to contain *Mtb*, their role is complex. Chronic inflammation can cause tissue damage, leading to healing processes like fibrosis that encapsulate the granuloma. While fibrosis limits tissue destruction, it also limits the infiltration of IFN*y*-producing immune cells, creating an environment that favors bacterial survival and sometimes replication. This imbalance can result in necrosis, cavitation, granuloma destabilization, rupture, and spread of *Mtb* within the lung, aiding transmission (17–21).

Mouse models have helped clarify granuloma behavior and mechanisms of disease progression. This study uses two mouse strains with distinct pathological outcomes. Balb/c mice form uniform, non-necrotic, non-encapsulated granulomas, supporting a slow, chronic infection similar to latent TB in humans. Conversely, C3HeB/FeJ mice develop heterogeneous lung lesions, including necrotic and encapsulated granulomas, with rapid disease progression and higher mortality (22). These differences allow comparison of granuloma immune structure across genetic backgrounds with varying control over *Mtb* (12, 13, 23).

Although necrotic granulomas have been well studied in mice and primates (12, 13, 15, 22), the transition from non-necrotic to necrotic granulomas is only beginning to be understood. Less attention has been paid to variation among non-necrotic granulomas across different mouse models. Here, we show that differences in the immune cell composition of non-necrotic granulomas in Balb/c and C3HeB/FeJ mice influence disease outcomes. Using advanced multiplex immunolabeling techniques, we examined the spatial cellular organization and interactions of myeloid and lymphoid cells. Our findings reveal significant differences in immune cell architecture, helping to explain why Balb/c granulomas tend to control disease better, whereas C3HeB/FeJ granulomas are associated with more severe pathology and faster progression.

## Materials and methods

### Mice and *Mtb* Infection

Female Balb/c and C3HeB/FeJ mice (6–8 weeks old) were obtained from Jackson Laboratories (Bar Harbor, ME). Upon arrival, mice were acclimated for at least one week in the BSL-3 facility at Colorado State University (CSU). All procedures were conducted in accordance with institutional guidelines, and all protocols were reviewed and approved by the CSU Institutional Animal Care and Use Committee (IACUC). The study used only female mice because long-term care of male mice infected with *Mtb* results in territorial and aggressive behaviors.

Mice were infected with a low-dose aerosol of *Mtb* Erdman strain (ATCC 35801) using a calibrated inhalation exposure system (Glas-Col, Terre Haute, IN) calibrated to deliver ~50–100 colony-forming units (CFU) to the lungs: ~100 CFU in Balb/c mice and 50–75 CFU in C3HeB/FeJ mice. To verify initial bacterial deposition, a subset of mice (n = 5) was euthanized 24 hours post-infection, and lung bacterial loads were quantified. The total CFU of the inoculum was determined by plating serial dilutions onto agar plates, as explained below. Mice were monitored daily for clinical signs of disease, including reduced activity, ruffled fur, hunched posture, and increased respiratory rate or effort.

### Necropsy of animals

*Mtb*-infected Balb/c mice (n = 5) were euthanized at 35-, 63-, and 77- days post-infection, whereas C3HeB/FeJ mice (n = 10) were euthanized at 63- and 91-days post-infection (1, 2). Mice were euthanized by CO_2_ narcosis, after which the thoracic cavity was opened, and the lungs were aseptically removed.

Because *Mtb* infection in Balb/c mice is uniformly distributed across lung lobes, only the left lung lobe (approximately one-third of total lung tissue) was collected for quantification of bacterial burden, while the right lung lobes were processed for histopathological analysis. In contrast, infection in C3HeB/FeJ mice is heterogeneous; therefore, whole lungs were collected for quantification of bacterial burden (n = 6 and n = 7), and lungs from a separate cohort (n = 3 and n = 4) were used for histopathology.

### Measurement of bacterial burden in the lungs

Lungs were homogenized using a Next Advance Bullet Blender (Averill Park, NY) as previously described (1, 2). Bacterial burden was quantified by plating serial dilutions of lung homogenates onto Middlebrook 7H11 agar and incubating at 37 °C. Colonies were counted after 4 weeks of incubation and reported as colony-forming units (CFU).

### Lung histopathology

Right lung lobes from Balb/c mice (n = 5) and whole lungs from C3HeB/FeJ mice (n = 3 and n=4) were fixed in 4% paraformaldehyde for 48 hours and embedded in paraffin (PFA-PE). These PFA-PE tissues were sectioned at 5 µm, dried overnight at room temperature, and stored at 4 °C until use (1, 2). Sections were stained with hematoxylin and eosin (H&E; Leica Biosystems) and scanned at 40× magnification using the automated multispectral PhenoImager™ HT system (Akoya Biosciences).

### Scoring lung lesions

Lung lesion burden was quantified on blinded digital images using QuPath software (3). H&E-stained sections were scanned at 40× magnification, and regions of interest (ROIs) were delineated using the Stamp feature. A custom tissue detection algorithm employing decision forest training and pixel classification distinguished lung tissue from non-tissue background. Automated cell segmentation based on hematoxylin staining was used to define individual cell boundaries. Lesion areas within tissue ROIs were identified using a custom algorithm that incorporates staining intensity, color normalization, deconvolution, area, and morphological features, generating heatmaps of cell density ranging from red (highest) to green (lowest), with lesion areas separated from uninvolved parenchyma by a dotted line. The percentage of lung tissue affected by *Mtb* (lesion) was quantified in QuPath by defining regions of interest (ROIs) and calculating the lesion area as a proportion of the total tissue area.

### Multiplex fluorescence immunohistochemistry

Five-micrometer PFA-PE lung sections were stained for multiplex fluorescence immunohistochemistry (mfIHC) using a Leica Bond autostainer. mfIHC was performed with an Opal seven-plex automation IHC kit (Quanterix) using the Tyramide Signal Amplification (TSA) technique. Two seven-plex antibody panels, together with DAPI nuclear staining, were developed as previously described (1), with detailed antibody and fluorophore information provided in **Supplementary Table 1**. Each antibody was optimized on a single-color IHC control slide using multiple mouse tissues. Based on fluorophore brightness, more abundant antigens were paired with less bright fluorophores, and TSA dilutions were titrated from 1:150. Full panels were validated prior to application to experimental lung tissue, with complete antibody and reagent details provided in **Supplementary Tables 1**, **2**. Moreover, *Mtb* staining using the OTB antibody on the consecutive lung section was done separately using Opal 570 fluorophore; however, its data was merged with panel 1 for analysis purposes.

### Image analysis and autofluorescence removal

mfIHC-stained sections were scanned at 40× magnification using the PhenoImager™ HT system for whole-slide imaging. Exposure times were optimized using the auto-exposure feature on representative ROIs from both murine models. Images were analyzed with Phenochart (v2.0.0) and inForm Tissue Finder (v2.4.8; Akoya Biosciences). Granuloma lesions and surrounding parenchyma were annotated using the Stamp feature, with at least five representative Stamps selected per tissue section (1). Tissue autofluorescence was removed using unstained control slides from the respective mouse strain. Artifacts such as tissue folds or fluorescence precipitates were excluded using the Disinterest Region feature. Thresholds for each fluorophore were adjusted to maintain consistent visualization. Batch analysis was performed for each time point and mouse strain, with quality control checks to remove unsuitable Stamps. Importantly, because the cell segmentation was based on DAPI staining, and staining of *Mtb* with OTB antibody was analyzed only by the intracellular method, leaving any extracellular staining unanalyzed.

### Data analysis and spatial quantification

Batch-analyzed Stamps were compiled into summary files and visualized using GraphPad Prism (v9.5.1) and R (v3.6.3). Spatial relationships of immune cells within granulomas were examined by plotting the X and Y coordinates of each phenotype using the phenoptrReports package in R. Nearest-neighbor distances were calculated to quantify interactions, and median distances from CD4+ and CD8+ T cells to other immune phenotypes were determined. Additionally, the average number of immune cells within a 100 µm radius of individual CD4^+^ and CD8^+^ T cells was calculated to assess local cell density and clustering.

### Statistical analysis

Bacterial burden data were log_10_-transformed and analyzed by one-way ANOVA with Tukey’s multiple-comparison test in GraphPad Prism. The percentage of granulomatous area, total immune cell counts, and cell densities were plotted in GraphPad Prism, with cell densities log_10_-transformed. Spatial metrics, including nearest-neighbor median distances and average cell counts within a 100 µm radius, were calculated from the X and Y coordinates obtained during image analysis and visualized as heatmaps in R using the ggplot package.

## Results

### Temporal changes in lung bacterial burden and granuloma pathology

**Figure 1:**
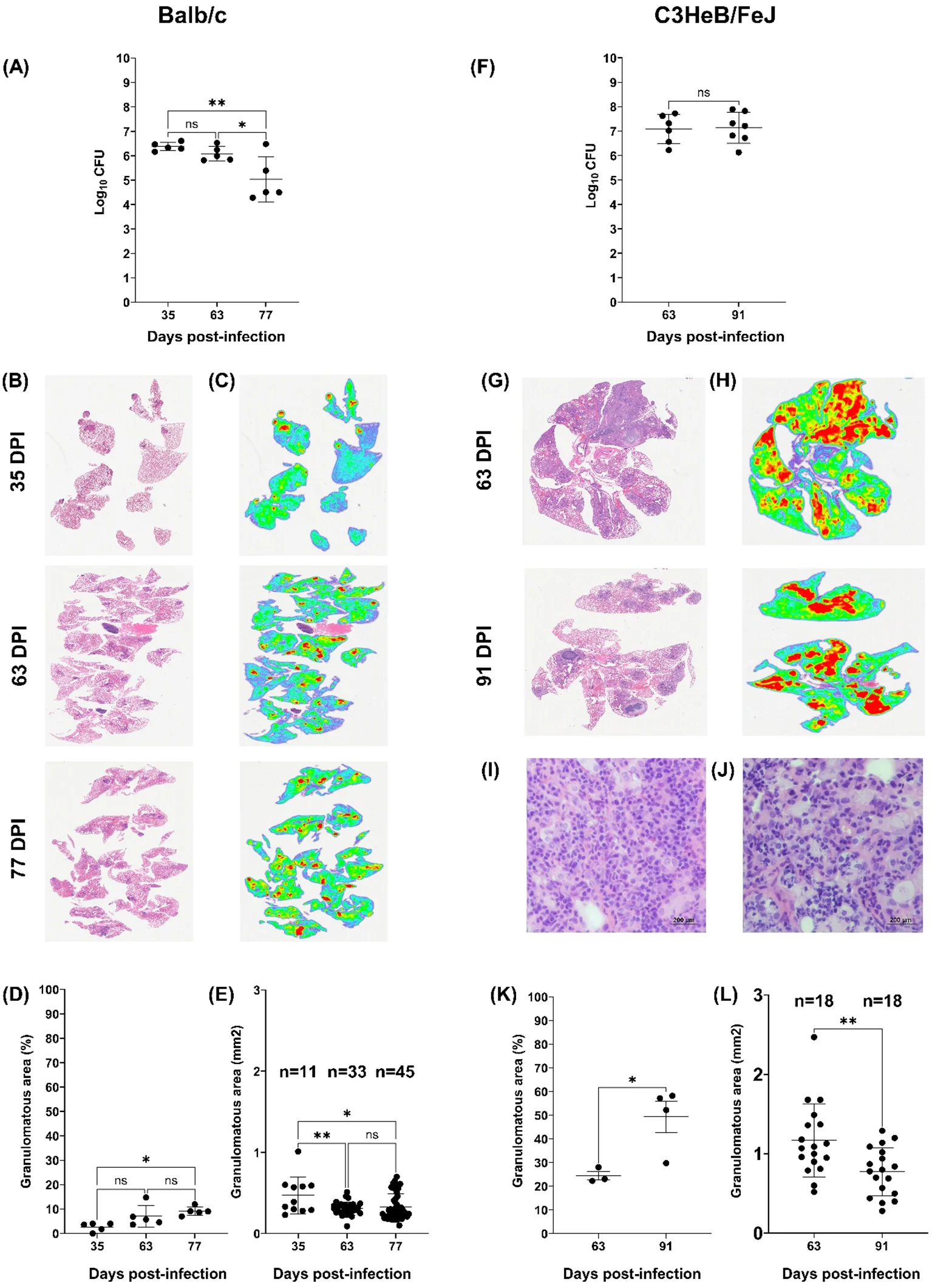
Lung bacterial burden and lesion quantification in Balb/c and C3HeB/FeJ mice infected with an aerosol of the *Mtb* Erdman strain. Lung bacterial burden (log_10_ CFU on the y-axis) in Balb/c mice **(A)** at 35, 63, and 77 days and in C3HeB/FeJ mice **(F)** at 63- and 91-days post-infection. Each dot in **(A, F)** represents a mouse. PFA-PE sections were cut at 5 µm, stained with Hematoxylin and Eosin (H&E), and imaged at 40× in Balb/c **(B)** and C3HeB/FeJ **(G)** and their corresponding heat maps are shown in **(C, H)**, respectively. The percentage of granulomatous area **(D, K)** in the respective mouse model) were calculated as the proportion of granuloma area over the total area of the lung section. H&E sections show the cellular structure of a non-necrotic granuloma in Balb/c **(I)** and C3HeB/FeJ **(J)** and their area of each granuloma is shown in **(E, L)**, respectively. The n values in **(E, L)** represent the total number of granulomas included for each time point. Statistical significance was calculated using one-way ANOVA with Tukey’s multiple comparison test. p < 0.05 was considered significant, and *p<0.01, **p<0.001, ns, not significant.

Twenty-four hours after aerosol challenge, Balb/c mice had approximately 100 CFU of *Mtb* (Erdman strain) in their lungs (data not shown). During the first 35 days post-infection (DPI), the pulmonary bacterial burden increased logarithmically to 6.37 ± 0.08 log_10_ CFU, then gradually declined to 6.08 ± 0.13 and 5.03 ± 0.41 log_10_ CFU at 63 and 77 DPI, respectively (**Figure 1A**). H&E-stained lung sections revealed the expected pathology in Balb/c mice (representative images shown in **Figure 1B**). To better visualize granulomatous lesions, cell density heatmaps were generated from H&E images (**Figure 1C**), highlighting granulomas (red), inflammation (green), and unaffected tissue (blue). The percentage of granulomatous area, calculated as the percentage of total lung area occupied by granulomas, increased from an average of 3% at 35 DPI to nearly 10% at 77 DPI (**Figure 1D**). The total number of granulomas identified (and analyzed in this study) at 35, 63, and 77 DPI was 11, 33, and 45, respectively (**Figure 1E**). Interestingly, while the total number of granulomas increased over time, the average area of individual granuloma decreased from 35 to 63–77 DPI (**Figure 1E**), suggesting granuloma proliferation with concomitant size reduction.

C3HeB/FeJ mice initially received approximately 50 CFU in the lungs (data not shown). Pulmonary bacterial loads increased logarithmically to 7.08 ± 0.24 and 7.13 ± 0.24 log_10_ CFU at 63 and 91 DPI, respectively, with no statistically significant difference between these two time points (**Figure 1F**). Despite implanting half as many CFUs in the lungs of Balb/c, C3HeB/FeJ mice exhibited approximately tenfold higher bacterial burdens at all time points. Representative H&E images and their corresponding cell density heatmaps are shown in **Figures 1G, H**, respectively. The percentage of granulomatous area increased significantly from 63 to 91 DPI (**Figure 1K**), whereas the area of individual granulomas decreased over time (**Figure 1L**). Notably, necrotic lesions were present in C3HeB/FeJ lungs by 63 DPI (**Supplementary Figure 1B, 1-2**). Comparison between strains revealed that both the percentage of granulomatous area and the granuloma area were three- to fivefold higher in C3HeB/FeJ mice than in Balb/c mice, highlighting the more severe and heterogeneous lung pathology in the former model.

Histopathological features of granulomas in H&E-stained sections from Balb/c mice were not markedly different from those of non-necrotic granulomas in C3HeB/FeJ (**Figures 1I, J**, respectively). In fact, a previous publication (12, 13) described non-necrotic granulomas (also referred to as type III lesions) in C3HeB/FeJ mice that were similar to those in Balb/c. In both instances, H&E-stained granulomas revealed mononuclear macrophages, some with a foamy appearance, and epithelioid macrophages intermixed with lymphocytes. Occasionally, small pockets of neutrophils (more abundant in C3HeB/FeJ mice) were present within lesions. Lymphocytes were seen both aggregated and scattered, interspersed with macrophages.

### Multiplex fluorescence immunohistochemistry reveals granuloma immune architecture

**Figure 2:**
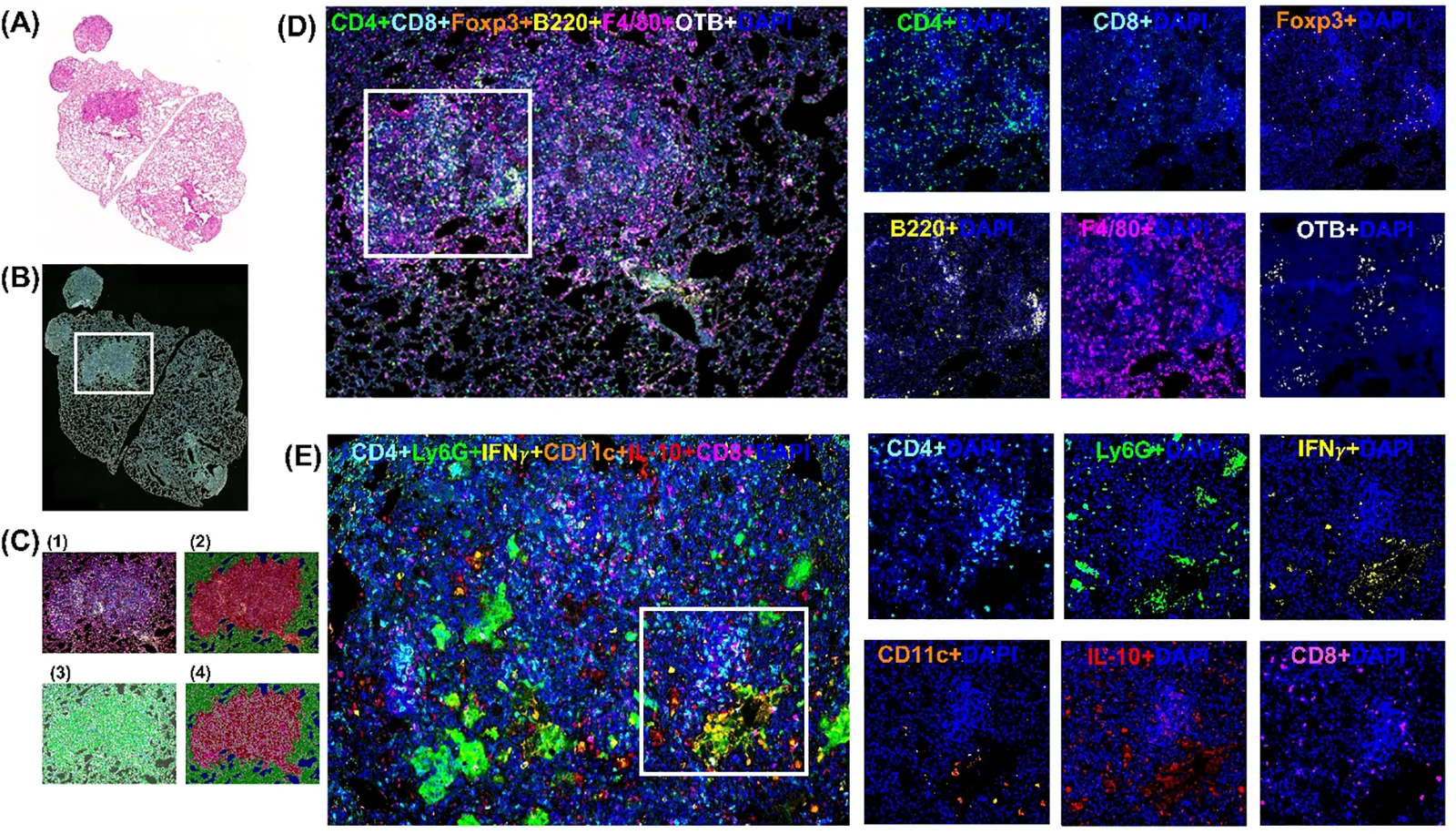
Multiplex fluorescence immunohistochemistry of lung tissue in TB mouse models. PFA-PE lung sections from Balb/c and C3HeB/FeJ TB mice were collected in Balb/c mice at 35, 63, and 77 DPI and in C3HeB/FeJ mice at 63- and 91-DPI, stained with two multiplex fluorescence immunohistochemistry (mfIHC) panels (each with 7-plex markers including DAPI). Representative H&E **(A)** and its corresponding mfIHC image **(B)** were taken from whole slide scans (**Supplementary Figure 2A**) at 40× magnification using PhenoImager. The workflow for digital pathology analysis of mfIHC image **(B)** was shown in **(C)** where a region of interest (ROI; white box in B) representing typical granuloma tissue selected using Phenochart software. The selected raw ROI was imported into inForm Tissue Finder software for image analysis using the 4 steps shown in **(C)**; the unmixing of raw images based on the spectral library (C1), the automated tissue segmentation based on training of algorithms for different tissue types (lesion in red, parenchyma in green, while blue depicts no tissue or blank slide region) (C2), the cell segmentation based on the DAPI marker (blue) (C3), and the quantification of immune cell phenotypes based on the specific fluorescence signal for each antibody (C4; F4/80+ staining in purple). ROIs **(D, E)** stained with two mfIHC panels; D = CD4 (green), CD8 (cyan), Foxp3 (orange), B220 (yellow), F4/80 (magenta) and OTB (white), while E = CD4 (cyan), Ly6G (green), IFN*y* (yellow), CD11c (orange) IL-10 (red) and, CD8 (magenta) where a large composite multicolor image shown on (left) and a subset (white box) of its corresponding individual single-color images for each antibody are shown on (right).

Lung sections from Balb/c and C3HeB/FeJ TB mouse models were collected at specific time points post-infection, as explained above, and stained with two seven-plex mfIHC panels, each including the nuclear stain DAPI. Details are summarized in **Figure 2** and **Supplementary Table 1**. Five-micrometer PFA-PE lung sections from each mouse model and control tonsil tissue were used to optimize primary antibody staining and refine the Opal seven-plex immunolabeling protocols. The panels included canonical myeloid and lymphoid markers, including T cell (CD4, CD8), B cell (B220), regulatory T cell (Foxp3), mycobacterial LAM antigen (OTB), macrophage (F4/80), neutrophil marker (Ly6G), dendritic cell (CD11c), and inflammatory and anti-inflammatory cytokines (IFN*y* and IL-10, respectively). Monoclonal antibodies and their corresponding fluorophores are detailed in **Supplementary Tables 2**, **3**.

**Figure 3:**
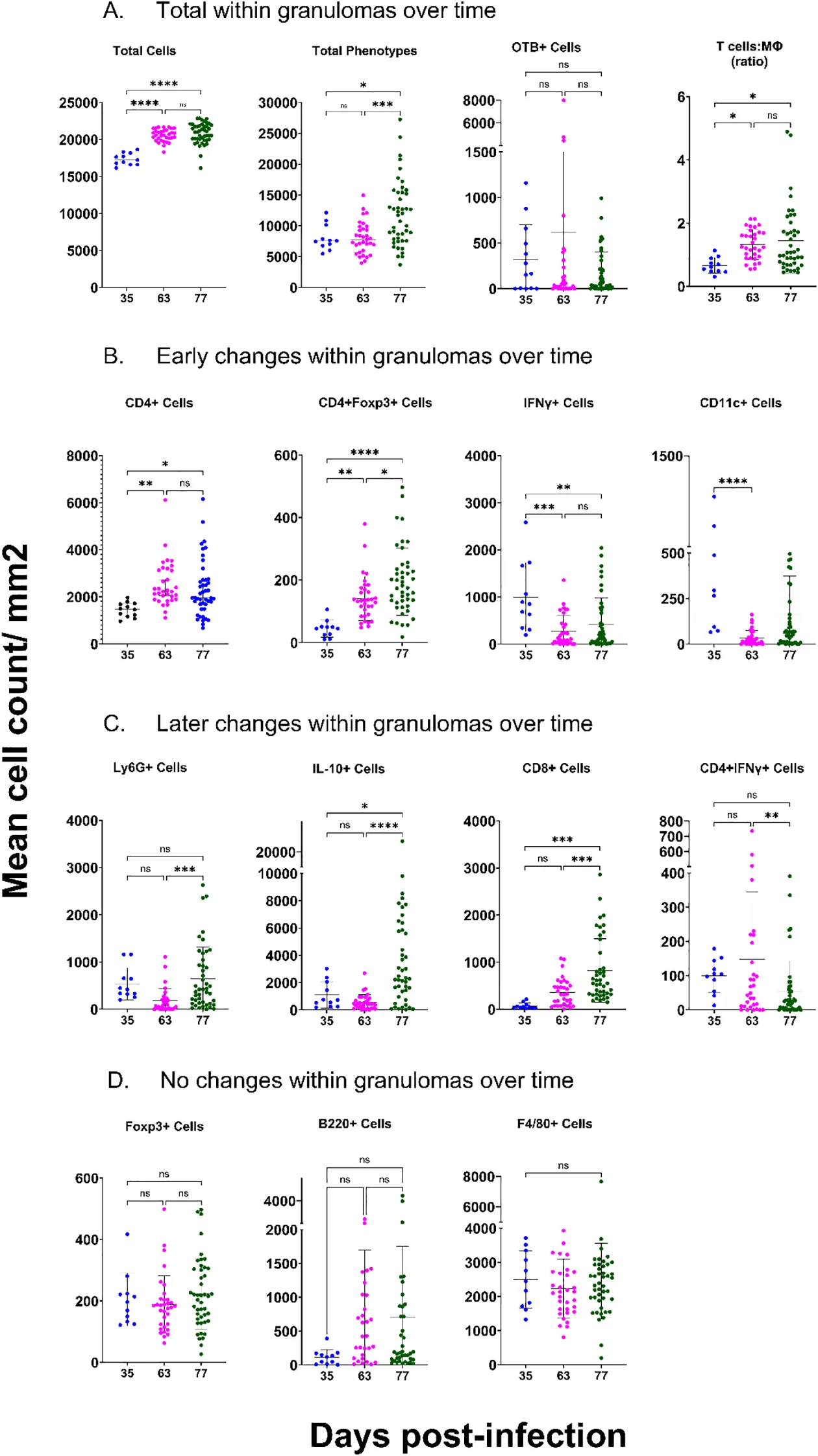
Temporal quantification of immune cells in lung non-necrotic granulomas of Balb/c mice. PFA-PE lung sections from Balb/c mice at 35, 63, and 77 DPI were stained with two mfIHC panels (each a 7-plex panel including DAPI), and image analysis was performed to quantify the temporal cell density of each immune cell per mm² of a granuloma. To normalize and compare granulomas, cell densities were calculated for each fluorescence marker (number of positive cells per mm² of a granuloma). Graphs in **(A)** represent Total Cells, which is the average total cell density within granulomas, Total Phenotypes is the total number of cells positive for at least one marker, OTB+ cells correspond to the quantification of mycobacterial lipoarabinomannan using the OTB antibody; and T cells: MΦ shows the ratio of the number of T cells to macrophages (MΦ). Graphs in **(B)** show early changes in cell number per mm² in the granuloma (35–63 DPI) for the cell markers CD4+, CD4+Foxp3+, IFN*y*+, and CD11c+. Graphs in **(C)** show later changes (63–77 DPI) for Ly6G+ neutrophils, IL-10+ cells, CD8+ T cells, and CD4+/IFN*y*+ double-positive cells. Graphs in **(D)** show the markers (Foxp3+ regulatory T cells, B220+ B cells, and F4/80+ macrophages) without changes throughout the infection. Each dot represents a cellular granuloma at each time point. Statistical significance was calculated using one-way ANOVA with Tukey’s multiple comparison test. p < 0.05 was considered significant, and *p<0.01, **p<0.001, ***p<0.0001, ****p<0.00001, ns, not significant.

**Figure 4:**
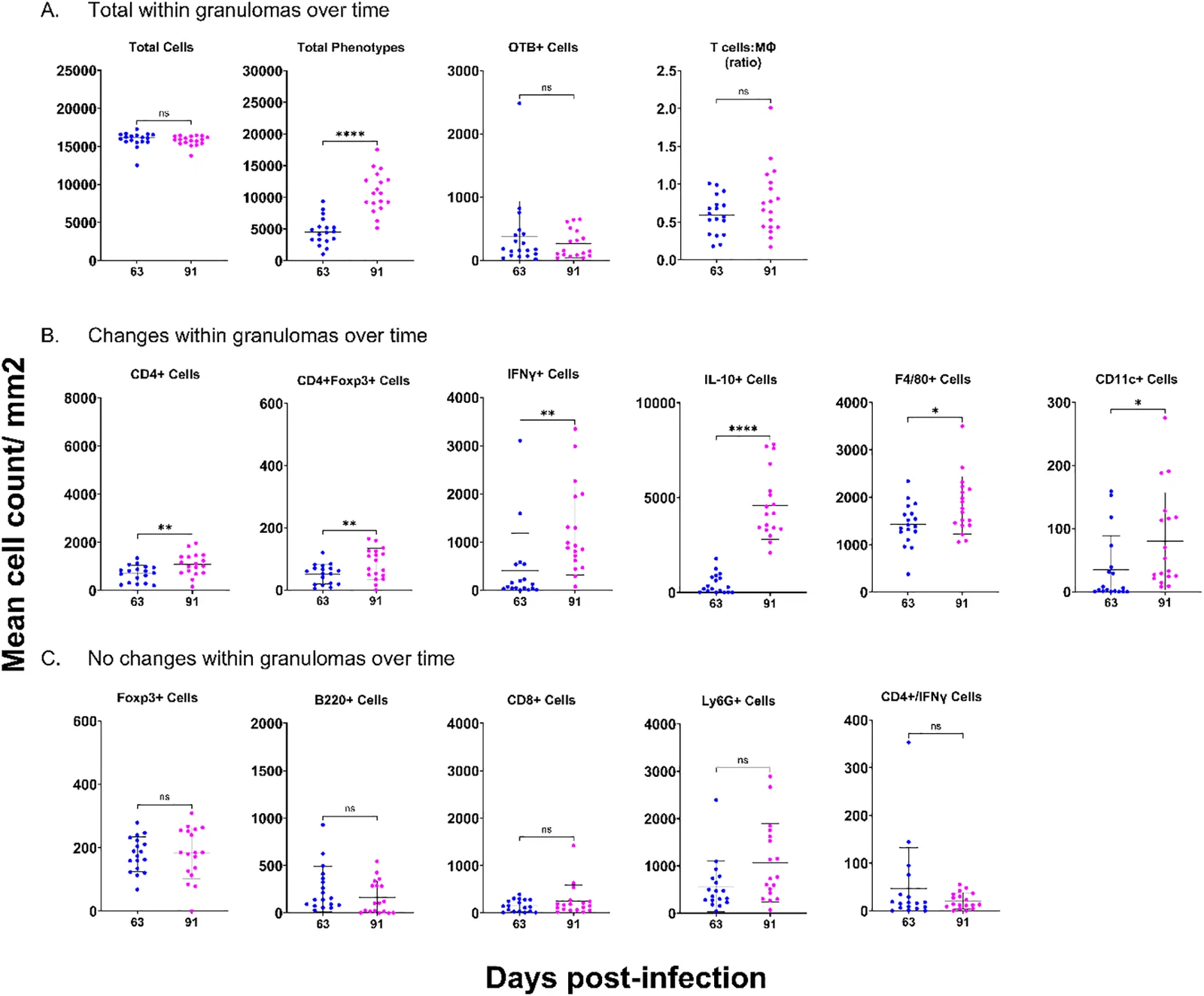
Temporal quantification of immune cells in lung non-necrotic granulomas of C3HeB/FeJ mice. The same mfIHC image analysis workflow used for Balb/c granulomas was applied to non-necrotic granulomas (n = 18) from C3HeB/FeJ TB mice at 63- and 91-days post-infection (DPI). Graphs in **(A)** show that Total Cells represent the average total cell density within granulomas at 63-91-DPI. Total Phenotypes represent the total number of cells positive for at least one marker. OTB+ cells quantify mycobacterial lipoarabinomannan using the OTB antibody, and T cells: MΦ shows the ratio of T cells to macrophages (MΦ). In **(B)**, graphs show changes in cell number per mm² of a granuloma (63–91 DPI) for the cell markers CD4+, CD4+Foxp3+ cells, IFN*y*+, IL-10+, F4/80+, and CD11c+. Graphs in **(C)** show markers that do not change over time: Foxp3+, B220+, CD8+, Ly6G+, and CD4+/IFN*y*+. Each dot represents a cellular granuloma at each time point. Statistical significance was calculated using one-way ANOVA with Tukey’s multiple comparison test. p < 0.05 was considered significant, and *p<0.01, **p<0.001, ***p<0.0001, ****p<0.00001, ns, not significant.

A standardized workflow was established for digital image analysis of the mfIHC-stained sections (**Figure 2**), with additional representative H&E and mfIHC whole-slide scan images shown in **Supplementary Figures 2A**, **3**. mfIHC-stained sections were scanned at 40× magnification using the PhenoImager™ HT system for whole-slide imaging (**Figures 2A, B**), and multiple ROIs, each representing a granuloma and its surrounding parenchyma, were selected using Phenochart software (white box in **Figure 2B**). Raw images from each ROI were imported into inForm Tissue Finder software for analysis following four sequential steps: unmixing of raw images based on a spectral library (**Figure 2C-1**), automated tissue segmentation differentiating lesions (red), parenchyma (green), and blank regions (blue) (**Figure 2C-2**), cell segmentation using the DAPI nuclear stain (blue) (**Figure 2C-3**), and quantification of immune cell phenotypes based on specific fluorescence signals (F4/80+ macrophages shown in purple) (**Figure 2C-4**).

**Figure 2D** illustrates the resulting composite multicolor image, with a white box highlighting a representative ROI (left), alongside single-color images for each antibody (right): CD4 in green, CD8 in cyan, Foxp3 in orange, B220 in yellow, F4/80 in magenta and *Mtb* staining using the OTB antibody in white. **Figure 2E** shows the second panel on a similar lung section, with CD4 in cyan, Ly6G in green, IFN*y* in yellow, CD11c in orange, IL-10 in red, and CD8 in magenta. This approach enabled simultaneous visualization and quantification of multiple immune cell populations within individual granulomas, providing insights into the spatial organization and potential interactions of lymphoid and myeloid cells in TB granulomas from both mouse models.

### Immune cell landscape and spatial organization in non-necrotic granulomas from Balb/c mice

We compared the landscape of immune cells in non-necrotic cellular granulomas of Balb/c mice over time (**Figure 3**). To normalize and compare granuloma cellular composition, cell densities were calculated for each fluorescence marker (number of positive cells per mm² of granuloma). In Balb/c mice, the average total cell density within granulomas increased from 35 to 63 DPI but plateaued between 63 and 77 DPI (**Figure 3A**; Total Cells). This pattern suggests local recruitment or proliferation of immune cells during early granuloma maturation, followed by stabilization at later stages. The total number of cells positive for at least one marker remained largely stable between 35 and 63 DPI, yet variability increased at 77 DPI (**Figure 3A**; Total Phenotypes). Granuloma heterogeneity, assessed as the standard deviation of cell densities, also increased over time, indicating a more variable cellular composition at later stages. Quantification of mycobacterial lipoarabinomannan (LAM) using OTB antibody staining revealed no significant changes over time (**Figure 3A**; OTB+ cells). The ratio of T cell markers to macrophages (MΦ) within granulomas increased from 35 to 63 DPI, but no significant changes were observed between 63 and 77 DPI (**Figure 3A**; T cell: MΦ).

Analysis of individual immune populations revealed dynamic changes over the course of infection. Early changes in granulomas (35–63 DPI) included increased densities of CD4+ and CD4+Foxp3+ cells, accompanied by reductions in IFN*y*+ and CD11c+ cells per mm² (**Figure 3B**). At later stages (63–77 DPI), Ly6G+ neutrophils, IL-10+ cells, and CD8+ T cells increased, whereas CD4+/IFN*y*+ double-positive cells declined (**Figure 3C**). The numbers of Foxp3+ regulatory T cells, B220+ B cells, and F4/80+ macrophages remained relatively stable across all time points, although considerable heterogeneity was observed between individual granulomas (**Figure 3D**). By 77 DPI, granulomas were highly heterogeneous, with some displaying low and others high densities of positive cells; the only exception was CD4+/IFN*y*+ double-positive cells, which were already variable at 35 DPI.

Spatial analysis of immune cell distribution revealed that most immune cells, including CD4+, CD8+, Foxp3+, IFN*y*+, IL-10+, and Ly6G+ cells, were primarily located within granulomas rather than in the surrounding parenchyma (data not shown). These cells were generally scattered as individual cells throughout the granuloma. In contrast, B220+ B cells were initially sparse at 35 DPI but gradually formed distinct clusters that became more pronounced by 77 DPI (**Supplementary Figure 2B**). Together, these data suggest that Balb/c granulomas undergo temporal changes in cellular composition and spatial organization, with early recruitment of T and myeloid cells, later enrichment of regulatory and inflammatory populations, and the progressive clustering of B cells, reflecting a dynamic but controlled immune microenvironment within non-necrotic granulomas.

### Immune cell landscape and spatial organization in non-necrotic granulomas from C3HeB/FeJ mice

The same mfIHC image analysis workflow used for Balb/c granulomas was applied to non-necrotic granulomas from C3HeB/FeJ TB mice at 63- and 91 DPI (N = 18; **Figure 4**). Across the time points, total cell counts per granuloma and the abundance of the *Mtb* marker OTB remained unchanged, indicating stable overall granuloma cellularity and bacterial presence (**Figure 4A**). However, the number of distinct Total Phenotypes per granuloma increased over time, suggesting diversification of the immune response (**Figure 4A**). The ratio of T cells to macrophages (**Figure 4A**, T cell; MΦ) was similar at both time points.

From 63 to 91 DPI, significant increases were observed in the densities of CD4+ T cells, CD4+/Foxp3+ regulatory T cells, IFN*y*+ cells, IL-10+ cells, F4/80+ macrophages, and CD11c+ myeloid cells (**Figure 4B**). In contrast, the densities of Foxp3+, B220+, CD8+, Ly6G+, and CD4+/IFN*y*+ double-positive cells remained relatively stable between these time points (**Figure 4C**).

Overall, these data suggest that non-necrotic granulomas in C3HeB/FeJ mice show progressive enrichment of multiple T cell and myeloid populations over time, despite stable total cellularity and bacterial load. This contrasts with Balb/c granulomas, in which total cell numbers plateau and immune cell composition becomes more heterogeneous over time, highlighting strain-specific differences in granuloma immune dynamics that may contribute to differential disease progression.

### Comparative analysis of non-necrotic granulomas in Balb/c and C3HeB/FeJ Mice at 63 DPI

**Figure 5:**
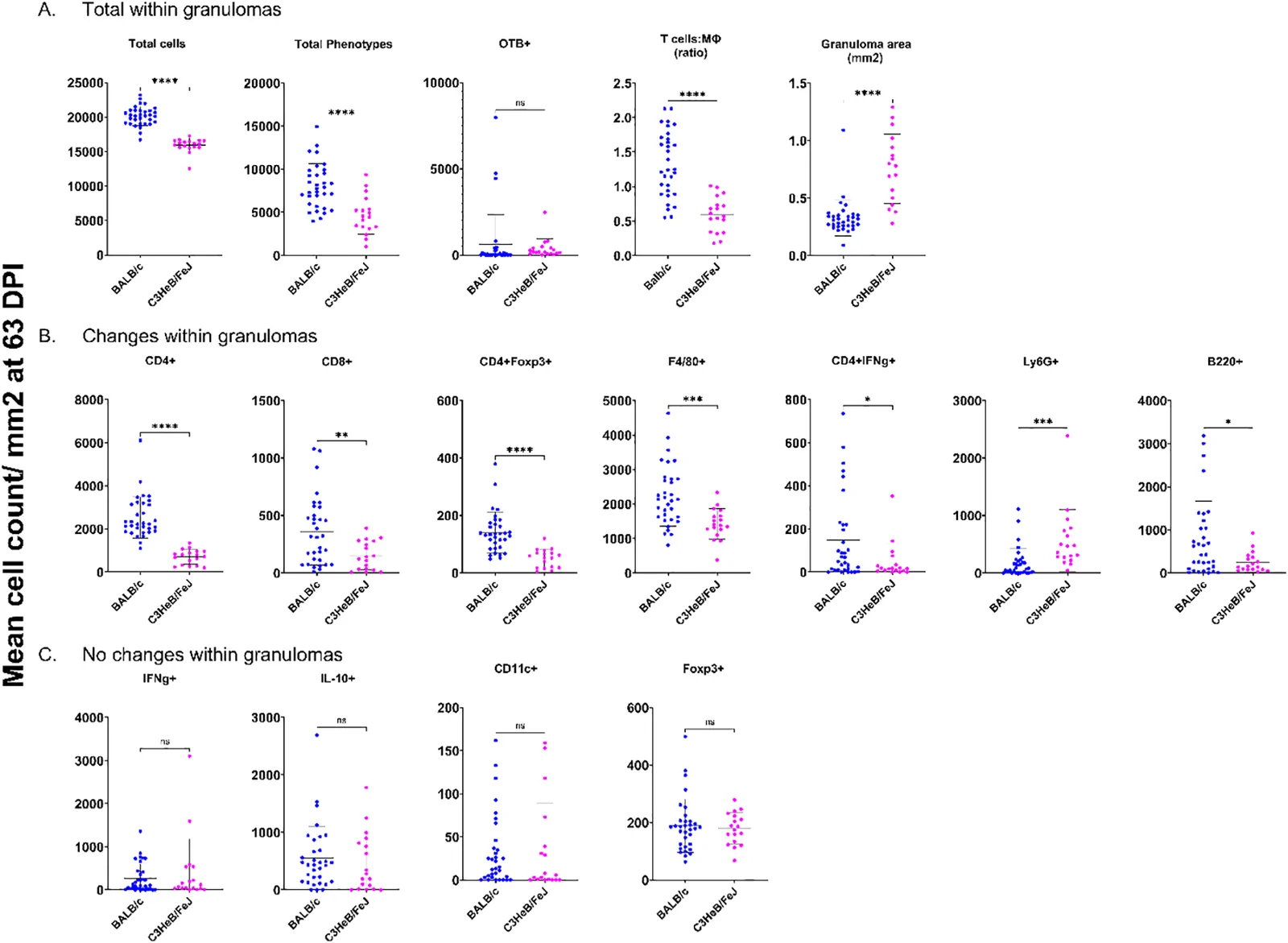
Comparison of immune cells in lung non-necrotic granulomas of Balb/c and C3HeB/FeJ mice at 63 days post-infection. To directly compare non-necrotic granulomas between the two TB mouse models, Balb/c granulomas (n = 33) and C3HeB/FeJ granulomas (n = 18) were analyzed at 63 DPI. **(A)** Graphs in the top row show Total Cells, defined as the average total cell density within granulomas. Total Phenotypes represent the total number of cells positive for at least one marker. OTB+ cells correspond to the quantification of mycobacterial lipoarabinomannan using the OTB antibody, and T cells: MΦ shows the ratio of the number of T cells to macrophages (MΦ). **(B)** The middle row shows changes in cell number per mm² of a granuloma for the cell markers like CD4+, CD8+, CD4+/Foxp3+, F4/80+, CD4+/IFN*y*+, LyG6+, and B220 +. **(C)** Graphs in the bottom row show markers that did not change between the two models: IFN*y*+, IL-10+, CD11c+, and Foxp3+. Each dot represents a cellular granuloma at each time point. Statistical significance was calculated using one-way ANOVA with Tukey’s multiple comparison test. p < 0.05 was considered significant, and *p<0.01, **p<0.001, ***p<0.0001, ****p<0.00001, ns, not significant.

To directly compare non-necrotic granulomas between the two TB mouse models, granulomas from Balb/c (n = 33) and C3HeB/FeJ (n = 18) mice were analyzed at 63 DPI (**Figure 5**). Although the average granuloma area (**Figure 5A**-Granuloma area, mm²) was larger in C3HeB/FeJ mice, Balb/c granulomas contained higher total cell densities (**Figure 5A**-Total cells) and exhibited greater phenotype diversity per mm² (**Figure 5A**-Total phenotypes). Interestingly, the abundance of Mtb mycobacterial marker-positive cells (OTB+) was similar across strains, indicating comparable levels of bacterial LAM antigen within granulomas (**Figure 5A**-OTB+). The T cell to macrophage number ratios were higher in Balb/c than in C3HeB/FeJ granulomas.

Comparative analysis of immune cell densities revealed that most T-cell and myeloid markers including CD4+, CD8+, CD4+/Foxp3+, F4/80+, CD4+/IFN*y*+, and B220+ cells were more abundant in Balb/c granulomas than in C3HeB/FeJ counterparts (**Figure 5B**). In contrast, Ly6G+ neutrophils were more numerous in C3HeB/FeJ granulomas. The densities of IFN*y*+, IL-10+, CD11c+, and Foxp3+ cells did not differ significantly between strains. These results indicate that, despite larger granuloma size, C3HeB/FeJ non-necrotic granulomas harbor fewer T cells and macrophages and a skewed immune composition, which may contribute to the differential control of bacterial replication and disease progression observed between the two mouse models.

**Figure 6:**
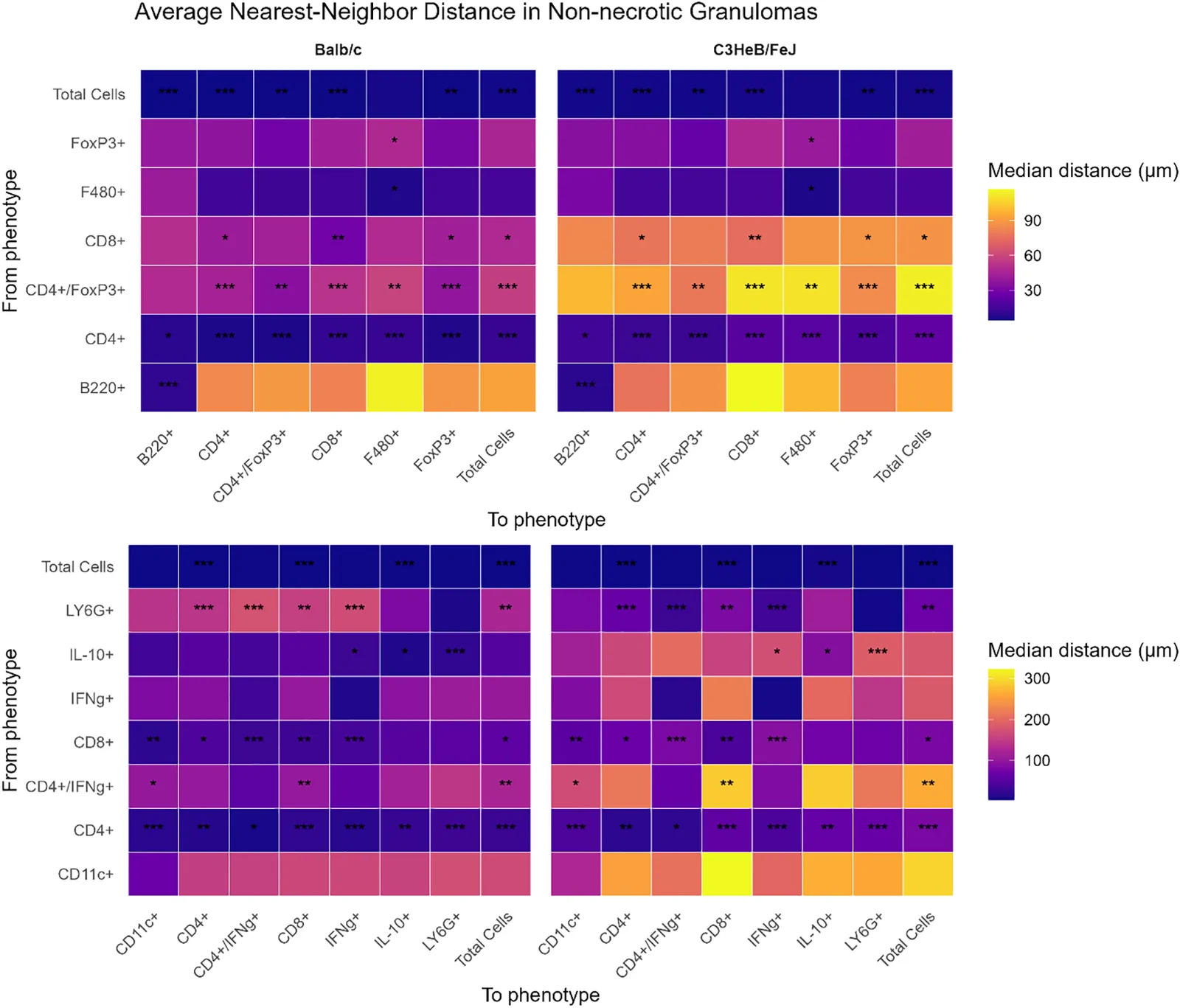
(**A** - Upper row) Comparison of nearest-neighbor distance of immune cells in lung non-necrotic granulomas of Balb/c and C3HeB/FeJ mice at 63 days post-infection. (**B** - lower row) Average median nearest-neighbor (NN) distances were calculated for immune cell phenotype pairs within non-necrotic granulomas from Balb/c and C3HeB/FeJ mice at 63 DPI. Heatmaps depict the mean of median NN distances (µm) for each phenotype pair, averaged across all granulomas per strain, with lower values indicating closer cell-cell proximity. Balb/c granulomas exhibit reduced NN distances between immune cells, consistent with a compact cellular architecture and coordinated immune interactions. In contrast, C3HeB/FeJ granulomas display increased NN distances, reflecting spatial disorganization despite increased granuloma size. Significant differences in NN distance between Balb/c and C3HeB/FeJ mice are marked with asterisks with a greater number of asterisks representing higher significance. However, NN distances involving Ly6G+ neutrophils show an opposite trend, with reduced distance in C3HeB/FeJ granulomas, consistent with neutrophil enrichment in progressive disease. Statistical comparisons between strains were performed using Wilcoxon rank-sum tests with false discovery rate (FDR) correction.

To assess how immune cell spatial organization differs between murine TB models, the median nearest-neighbor (NN) distances between immune cell phenotypes within non-necrotic granulomas were quantified (**Figure 6**). Median NN distance was selected as a robust spatial metric because it captures local cell–cell proximity while reducing sensitivity to outliers common in heterogeneous granulomatous lesions.

Balb/c granulomas consistently exhibited shorter median NN distances between adaptive and innate immune populations. In particular, CD4+ T cells were positioned in close proximity to F4/80+ macrophages and CD11c+ antigen-presenting cells, a spatial arrangement known to support efficient antigen presentation and IFN*y*-mediated macrophage activation during TB infection (5, 9, 10). CD8+ T cells and Foxp3+ regulatory T cells were also located closer to macrophages and neighboring immune cells, consistent with a compact, highly interactive granuloma microenvironment (**Figure 6A**).

In contrast, granulomas from C3HeB/FeJ mice showed significantly greater median NN distances across multiple phenotype pairs, suggesting a more dispersed cellular organization. Increased separation between CD4+ T cells and macrophages suggests impaired immune synapse formation and reduced functional coordination, which may contribute to the diminished bacterial control characteristic of this model. Neutrophil-rich granulomas in C3HeB/FeJ mice were also characterized by increased NN distances, indicative of spatially unstructured inflammation. (**Figure 6B**).

Comparative heatmaps of mean and median NN distances showed that, despite comparable mycobacterial antigen (OTB+) signals, Balb/c granulomas maintained significantly closer intercellular spacing than C3HeB/FeJ granulomas. These differences were statistically significant across multiple phenotype pairs, highlighting strain-dependent divergence in granuloma architecture rather than in bacterial burden alone.

## Discussion

Tuberculosis (TB) granulomas reflect a dynamic interplay between host immunity and *Mycobacterium tuberculosis* (*Mtb*) survival strategies. Previous study (4) showed that non-necrotic TB granulomas in Balb/c and C3HeB/FeJ TB mice are not grossly distinguishable on H&E sections, with similar overall architecture in both mouse strains. However, given the divergent disease trajectories and bacterial control between mouse strains, conventional histopathology alone did not clearly distinguish granulomas that contain *Mtb* without apparent contribution to disease progression from those that contribute to disease progression, underscoring the need for quantitative and spatial immune profiling to reveal functional differences in granuloma organization. In the present study, we compared the immune landscape of non-necrotic granulomas in Balb/c and C3HeB/FeJ murine TB models, revealing distinct temporal and spatial patterns that correlate with differential bacterial control and disease progression.

Our findings demonstrate that Balb/c granulomas, despite being smaller than C3HeB/FeJ granulomas, maintain higher total cell densities and greater phenotypic diversity per mm². These granulomas exhibit early recruitment of CD4+ and CD4+/Foxp3+ T cells, followed by later increases in Ly6G+ neutrophils, IL-10+ cells, and CD8+ T cells, while B220+ B cells progressively organize into localized clusters. This temporal orchestration suggests a controlled, adaptive immune environment that can restrict bacterial replication, consistent with the observed reduction in pulmonary bacterial burden over time. A similar temporal compartmentalization of adaptive and regulatory responses has been described in granulomas that contain disease progression in both murine and primate TB models (5, 17–20, 24). Importantly, the spatial organization of immune cells within Balb/c granulomas—characterized by dense localization of T cells and macrophages within the granuloma core—likely facilitates effective antigen presentation and local cytokine signaling. Close proximity between CD4+ T cells and macrophages is essential for IFN*y*-mediated macrophage activation and intracellular control of *Mtb* (9, 10, 24). The reduced median nearest-neighbor (NN) distances observed in Balb/c granulomas quantitatively support the presence of tightly organized immune networks that promote sustained effector function and bacterial containment.

In contrast, non-necrotic granulomas in C3HeB/FeJ mice showed progressive enrichment of multiple T cell and myeloid populations without a concomitant reduction in bacterial burden. These granulomas were larger, had lower overall cell densities, and exhibited reduced phenotypic diversity, suggesting a permissive microenvironment for *Mtb* persistence. The increased abundance of Ly6G+ neutrophils, combined with a dispersed spatial distribution of T cells and macrophages, indicates an inflammatory yet poorly coordinated immune response. This pattern is consistent with prior studies showing that neutrophil-dominated granulomas are associated with tissue damage, impaired bacterial control, and progression toward necrosis rather than protection (1, 16, 25–28). The negative correlation between immune cell densities and *Mtb* antigen expression (OTB+), although not statistically significant, further supports the idea of incomplete bacterial control despite immune cell recruitment.

Comparative analyses of the two mouse strains at 63 days post-infection revealed that, despite similar OTB+ bacterial antigen loads in non-necrotic granulomas, immune organization differed markedly. Balb/c granulomas were enriched for dense T cell–macrophage networks, whereas C3HeB/FeJ granulomas showed increased neutrophil representation and reduced immune cell coupling. These findings reinforce the concept that granuloma size or antigen burden alone does not predict bacterial control. Instead, immune cell density, phenotypic composition, and spatial arrangement are critical determinants of granuloma efficacy.

Analysis of average median NN distances provides a quantitative framework linking granuloma function to spatial immune organization. Short NN distances in Balb/c granulomas reflect compact immune architectures that enhance cellular communication, antigen recognition, and effector coordination. Conversely, increased NN distances in C3HeB/FeJ granulomas indicate spatial uncoupling of key immune interactions, including those between T cells and macrophages, which likely limits effective bacterial killing. Increased NN distances involving CD8+ T cells may further compromise cytotoxic surveillance, while neutrophil clustering may exacerbate inflammation without conferring protection.

These observations align with studies in non-human primates and humans showing that TB with disease progression is characterized by granulomas with structured immune niches and tight clustering of immune cells, whereas in cases of progressive TB disease, granulomas display spatial disorganization, neutrophil accumulation, and impaired immune coordination (14, 27–29). Collectively, our data support a model in which short nearest-neighbor distances between adaptive immune cells and macrophages are a hallmark of granulomas associated with TB without disease progression, whereas increased intercellular spacing contributes to immunopathology and disease progression.

Variations among granuloma types reflect known differences in macrophage susceptibility to *Mtb* infection across mouse strains. Macrophages from C3HeB/FeJ mice are more susceptible to *Mtb* than those from Balb/c mice (30, 31). C3HeB/FeJ mice are notably prone to *Mtb* infection, and their high susceptibility has been linked to the sst1 locus on chromosome 1 (3, 22, 30). These mice also have a unique immune profile, with differences in the regulation of macrophage apoptosis and necrosis during infection. Thus, the contrasting architectures observed in Balb/c and C3HeB/FeJ murine TB models provide insight into how host determinants of macrophage phenotype and the resulting immune differences during infection can influence susceptibility and disease progression. The result is that Balb/c granulomas exhibit features consistent with a controlled or latent-like infection, including compact cellular organization and effective T cell–macrophage interactions (18–20, 22, 24, 25, 28, 31), whereas the more heterogeneous immune landscapes of C3HeB/FeJ granulomas—characterized by increased neutrophil representation and disrupted spatial coordination—mirror those of progressive human tuberculosis (13, 23, 25, 26, 32, 33).

In conclusion, our findings highlight the importance of the temporal and spatial dynamics of immune cells in shaping granuloma function. Quantitative spatial metrics, such as nearest-neighbor distance, provide a powerful framework for assessing granuloma efficacy beyond traditional measures of size and cellular abundance. Understanding how immune cell organization governs granuloma stability and *Mtb* control may inform host-directed therapies, vaccine strategies, and biomarkers of disease progression (34–36). Future studies integrating functional assays and longitudinal spatial imaging will be critical for elucidating the mechanisms linking immune architecture to TB outcomes.

Several limitations of this study should be considered. Direct long-term comparisons between strains were constrained by the absence of 91- and 35-day post-infection time points in the Balb/c and C3HeB/FeJ TB models, respectively, underscoring the need for longitudinal studies with matched sampling to define late-stage granuloma dynamics. In addition, OTB immunohistochemistry detects mycobacterial antigen rather than viable bacilli. Because our analysis was restricted to intracellular OTB identified by DAPI-based segmentation, extracellular or non-replicating bacterial populations may be underrepresented. Newer methodologies, such as RNAscope, can be used in similar studies to quantify bacterial abundance within granulomas. Finally, immune cell phenotypes, densities, and spatial relationships were quantified using mostly single-marker expression; functional states such as cytokine production, macrophage activation, and bacterial viability were not directly assessed. Future studies integrating spatial analysis with functional and viability readouts will be essential to establish mechanistic links between immune architecture and granuloma efficacy.

## Data Availability

The raw data supporting the conclusions of this article will be made available by the authors, without undue reservation.
