# Supplementary material for: Spatial and temporal immune landscapes of non-necrotic granulomas in two murine tuberculosis models with divergent disease outcomes
Source: Front Immunol. 2026 Aug 13;17:1821629. doi: 10.3389/fimmu.2026.1821629 (PMC13519991; doi:10.3389/fimmu.2026.1821629)
Supplement: Supplementary file 1 [file SupplementaryFile1.pdf]

Supplementary data

Spatial and Temporal Immune Landscapes of Non-Necrotic Granulomas in Two Murine Tuberculosis Models with Divergent Disease Outcomes

Malik Zohaib Ali[1, 2, 3], Taru S. Dutt [4], Johnathan Patterson[1,2, *], Angela Minic [5], Kimberly R. Jordan[5], Sara E. Maloney Norcross [6], Bernd Meibohm[7], Mercedes Gonzalez-Juarrero[1, 2, 3 **]

[1]Mycobacteria Research Laboratories, Colorado State University, Fort Collins, CO, 80523, USA

[2]Microbiology, Immunology and Pathology, Colorado State University, Fort Collins, CO, 80523, USA

[3] Program in Cell & Molecular Biology, Colorado State University, Fort Collins, CO, 80523, USA

[4]Department of Pathology, Heersink School of Medicine, University of Alabama at Birmingham, Birmingham, 35233, USA

[5]Department of Immunology and Microbiology, University of Colorado Anschutz Medical Campus, Denver, CO, 80045, USA

[6]Engineering and Advanced Technology Department, RTI International, Durham, NC, 27713, USA

[7]Department of Pharmaceutical Sciences, University of Tennessee Health Science Center, Memphis, TN, 38163, USA

* Current address: National Jewish Health; Department of Immunology and Genomic Medicine, Denver, CO, 80206, USA

Keywords: spatial analysis, multiplex fluorescence immunohistochemistry, non-necrotic granuloma, *Mycobacterium tuberculosis*, immune cells, C3HeB/FeJ, Balb/c mice, inForm Tissue Finder

Running title

Immune landscapes of non-necrotic granulomas with different disease outcomes.

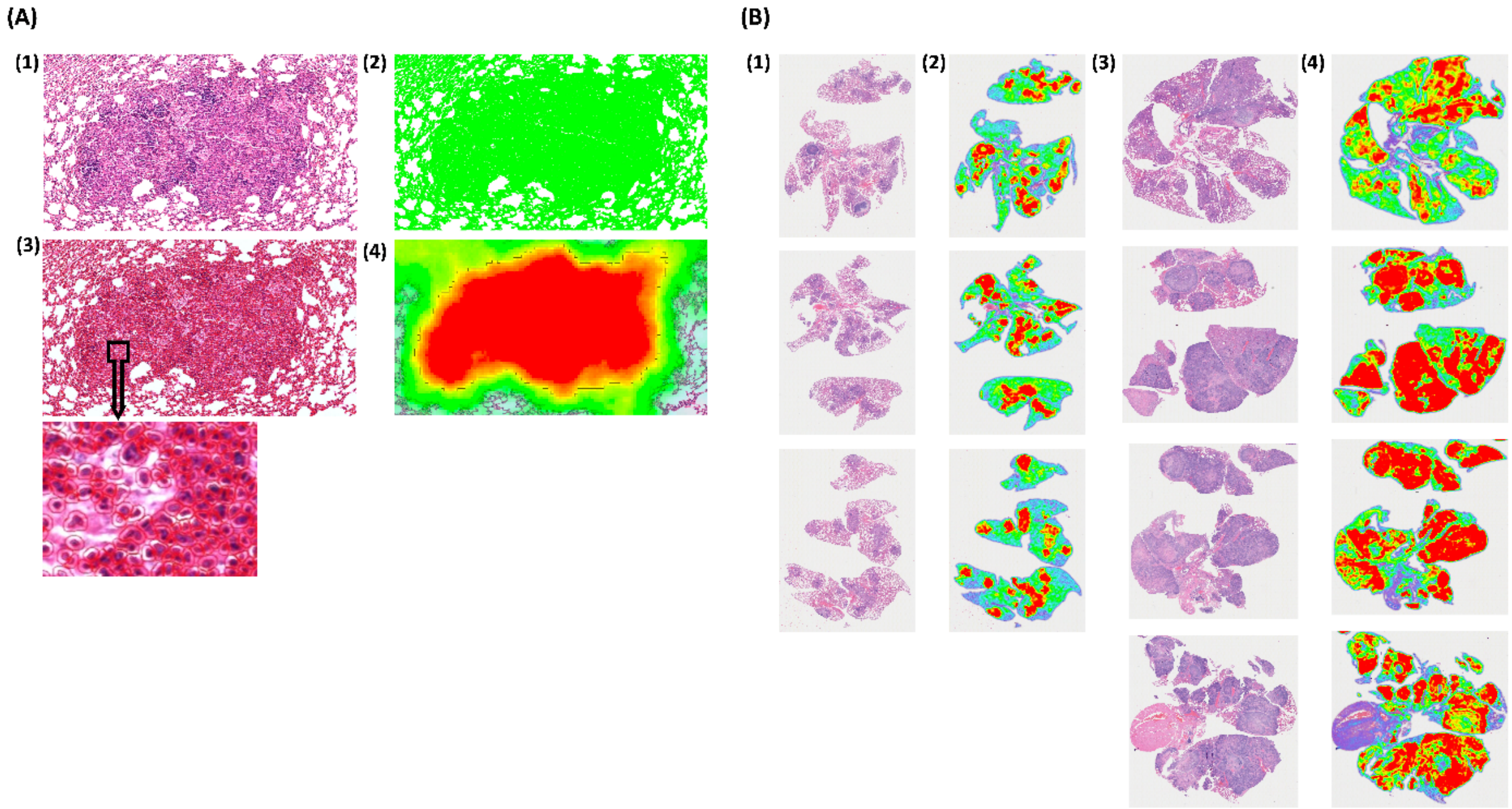

Supplementary Figure 1. (A) describes step-by-step protocol for lesion quantification using an open source QuPath software. (1) H&E image scanned at 40×, (2) Thresholding based on pixel classification to select and differentiate lung tissue (green) from non-tissue background (white), (3) Cell segmentation for further analysis based on Hematoxylin staining (insert represents a magnified view of cell boundaries), (4) Heatmaps for cell density shown as a grading from red (highest) to green (lowest) density. A black dotted line differentiates lesion area (red) from uninvolved parenchymal tissue (green). (B) Whole-slide scans from C3HeB/FeJ mice stained with H&E (1,3) and their respective lesion density maps (2,4) at 63 (n = 3) and 91 (n = 4) DPI, respectively.

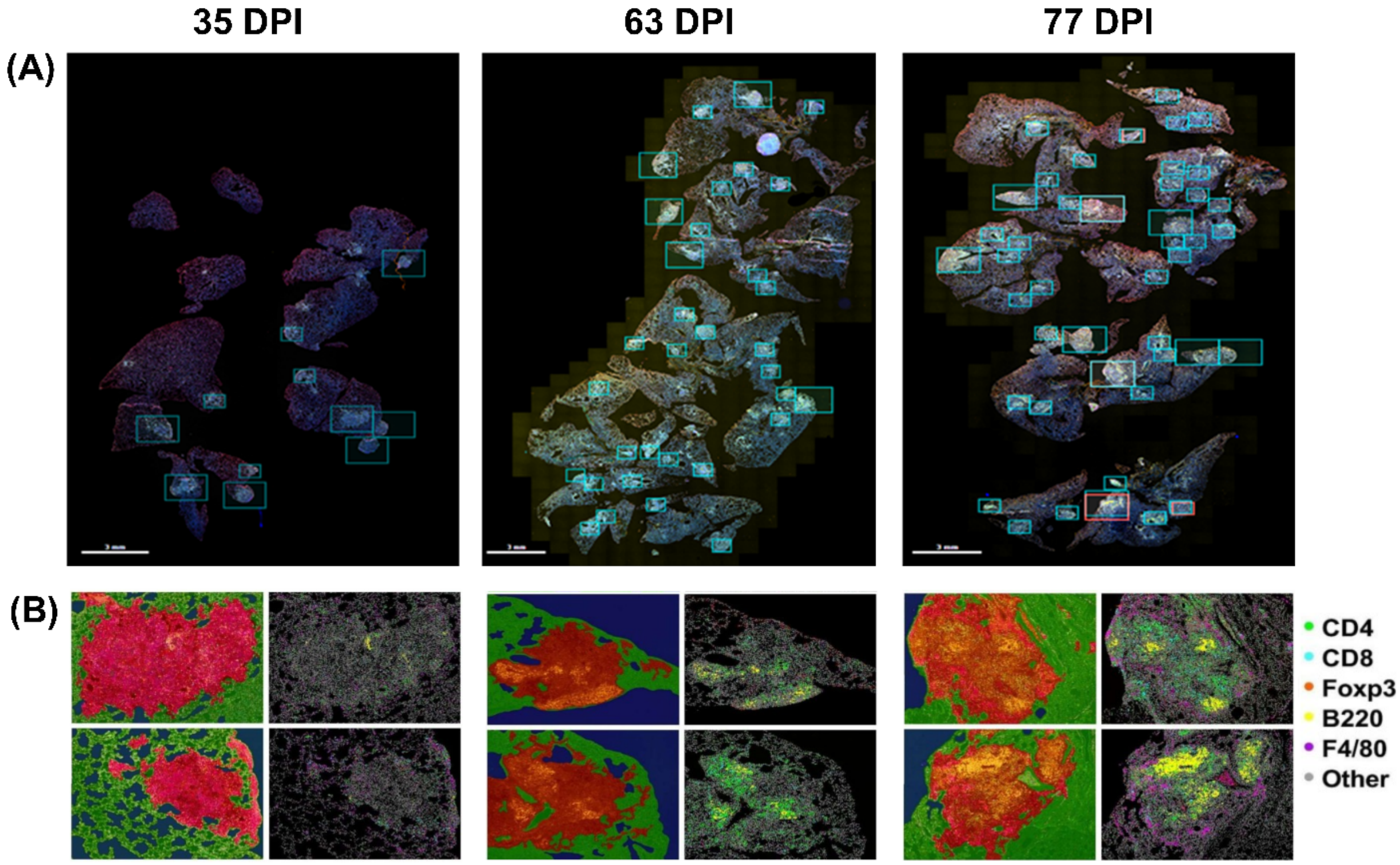

Supplementary Figure 2. Whole-slide scans of lung sections from Balb/c TB model at 35, 63, and 77 DPI (A). Slides were stained with two mfIHC panels as explained in Figure 2. Green boxes indicate the annotations for selected non-necrotic granulomas for analysis. 40x magnification. (B) Each time point represents the granuloma structure in red and parenchyma in green on the left panel while the panel on the right represents immune markers indicated with different colors.

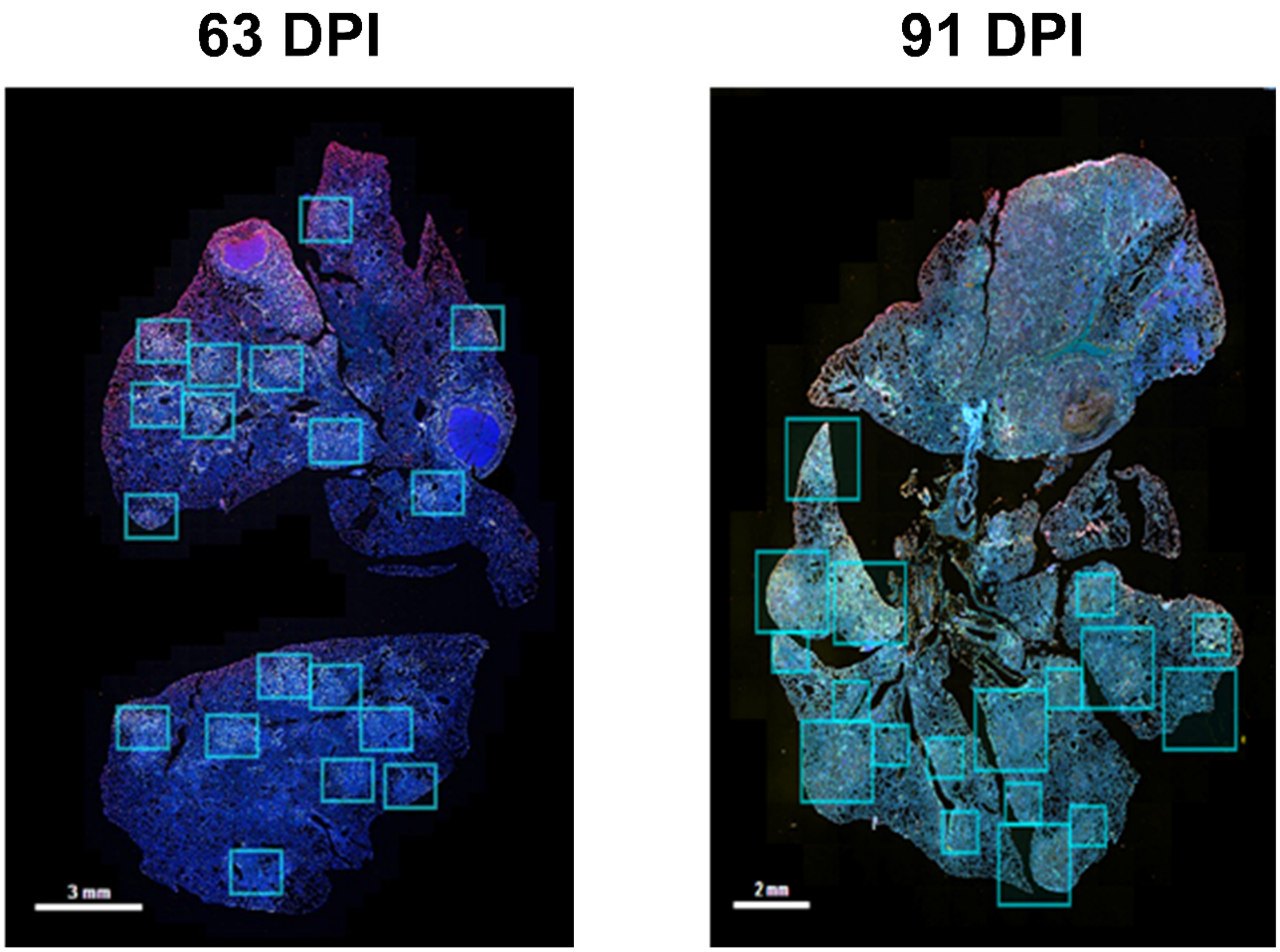

Supplementary Figure 3. Whole-slide images of lung sections from the C3HeB/FeJ TB model at 63 and 91 DPI. Slides were stained with two mfIHC panels as described in Figure 2. Green boxes indicate the selected cellular granulomas for analysis. 40x magnification.

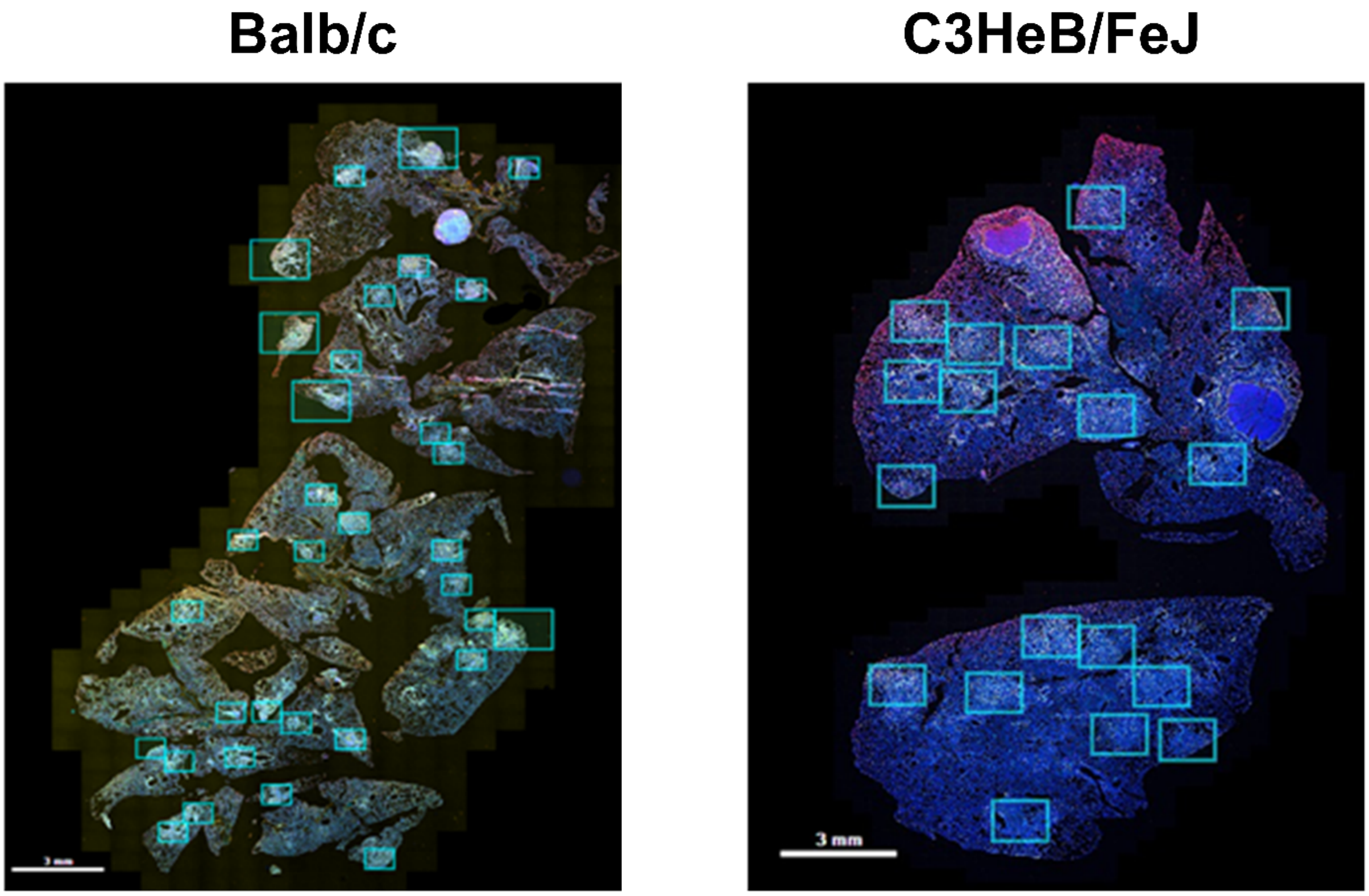

Supplementary Figure 4. Whole-slide images of lung sections from Balb/c and C3HeB/FeJ TB models at 63 DPI. Slides were stained with two mfIHC panels as described in Figure 2. Green boxes indicate the selected granulomas for analysis. 40x magnification.

Supplementary Table 1. Details on the antibodies combine with their respective fluorophore in each of 6-color plus DAPI mfIHC staining panel.

Panel	Opal fluorophore						
	**480**	**520**	**570**	**620**	**690**	**780**	**Nuclear staining**
1	CD8	CD4	B220	Foxp3	OTB (570)	F4/80	DAPI
2	CD4	Ly6G	IFNγ	CD11c	IL-10	CD8	DAPI

Supplementary Table 2. Details of the antibodies used in mfIHC staining.

Phenotype _Antibody	Specie	Type	Company	Cat#	Clone/ Lot#	Concentration	pH
CD4	Rat	Monoclonal	Thermo Fisher	740028	4SM95	1:200	6
CD8	Rabbit	Monoclonal	CST	98941	D4W2Z	1:400	6
B220	Rat	Monoclonal	BD Biosciences	14-0452-82	RA3-6B2	1:500	6
Foxp3	Rabbit	Monoclonal	R&D	MAB8214	1054C	1:200	9
F4/80	Rabbit	Monoclonal	CST	30325	D4C8V	1:100	6
CD11c	Rabbit	Monoclonal	CST	97585S	D1V9Y	1:300	6
Ly6G	Rabbit	Monoclonal	CST	87048	E6Z1T	1:100	6
IFNγ	Rabbit	Polyclonal	Bioss	bs-0480R	BJ07074740	1:200	6
IL-10	Rabbit	Polyclonal	Bioss	bs-20373R	BA01279056	1:200	6
OTB	Rabbit	Monoclonal	Otsuka	-	-	1:100	6

Supplementary Table 3. Brightness ranking of Opal fluorophores.

Opal fluorophore	Brightness ranking
Opal 480	Highest
Opal 520	Highest
Opal 570	Medium
Opal 620	Medium
Opal 690	Lowest
Opal 780	Lowest
